# Cascaded Safety Analysis and Test Scenario Generation Techniques for Autonomous Driving: A Case Study with WATonoBus

**DOI:** 10.1007/s42154-024-00313-z

**Published:** 2025-03-28

**Authors:** Chen Sun, Ruihe Zhang, Ahmad Reza Alghooneh, Minghao Ning, Pouya Panahandeh, Steven Tuer, Amir Khajepour

**Affiliations:** Department of Mechanical and Mechatronics Engineering, University of Waterloo, Waterloo, ON N2L 3G1 Canada

**Keywords:** Safety verification, Autonomous driving, Test case generation

## Abstract

Efficient exploration and understanding of an autonomous driving system’s capabilities and functional boundaries are crucial for ensuring safety performance. This paper offers a comprehensive examination of safety verification and test case generation for autonomous driving function stacks, enhancing their safety and reliability. Firstly, we introduce a holistic approach that synergizes operational flow-oriented Hazard and Operability Study (HAZOP) with cascaded System-Theoretic Process Analysis (STPA) processes. Secondly, we propose a test case generation procedure that begins with an expansion to discrete parameters using tree search, followed by heterogeneous sampling in the continuous parameter space. Additionally, this paper features a real-world case study with WATonoBus, showcasing the practicality and effectiveness of the proposed methods in securing autonomous vehicles safe operation in complex urban settings. Our findings make a substantial contribution to the autonomous vehicle safety field, offering critical insights for ongoing research and development in this rapidly advancing area.

## Introduction

Autonomous driving services possess unique characteristics such as automated driving, operation within restricted Operational Design Domains (ODD), regulated speeds, and shared services [1]. These services, particularly the last-mile public transportation, have garnered significant interest due to their less challenging service environment. Safety is paramount in autonomous driving services, necessitating rigorous testing and validation processes to ensure functionality, integration, and interaction of subsystems [2]. Enhancing the adaptability of autonomous vehicles to diverse and unpredictable real-world scenarios remains a significant challenge despite advancements.

The complexity of Automated Driving Systems (ADSs) poses challenges in assessing risk and safety, especially in services interacting with passengers and pedestrians. Accidents in ADSs underscore the need for innovative testing methods [3]. Safety validation often relies on scenarios, foundational to hazard and fault tree analysis, Scenario-Based Testing (SBT), and evaluation [4–6]. Prioritizing scenario quality over quantity in ADS validation is crucial due to the impracticality of achieving extensive real-world testing. Improving scenario quality involves exploring unknown details within the known ODD.

To assert the safety and robustness of ADS within the ODD, it’s essential to identify potential hazards, create comprehensive test scenarios covering these hazards, and express safety levels with stopping criteria. However, challenges persist in linking hazard analyses to the ADS’s ODD and defining accurate scenarios. The question of “how safe is safe enough?” remains a longstanding challenge in the autonomous driving industry. In this paper, we aim to address the aforementioned research questions. The major contributions of this paper are: (1) A methodology that integrates HAZOP and STPA within the ADS’s ODD context, setting the foundation for validation scenarios and constraints on scenario parameter search spaces. (2) A hybrid approach for generating test scenarios at an abstract level is proposed, incorporating a discrete parameter expansion tree alongside continuous search spaces. This approach focuses on perception uncertainty while omitting the physical or illustrative context of driving scenarios. A theoretical analysis of safety arguments and pass criteria is also presented. (3) A case study with a real autonomous shuttle bus, WATonoBus [7], focusing on verification in specific tasks deployed on the University of Waterloo Ring Road.

The following sections summarize recent studies in system verification for autonomous driving and methods for test case generation. In Sect. 3, we introduce our methodology, extending HAZOP and STPA to identify potential hazards for ADS and linking them to scenario generation parameters within the existing ODD. We emphasize the proposed test case generation procedure and our approach to deciding passing criteria for safety evaluation. In Sect. 4, we present a real-world case study on the safety validation process for ADS functions in WATonoBus, a Level 3 autonomous shuttle bus. Section 5 discusses the proposed approach and test results with WATonoBus, while Sect. 6 concludes the paper.

## Related Works

### Safety Analysis for Autonomous Driving Services

Despite the potential benefits of autonomous driving services, particularly in public transport and economic advantages, ensuring safety performance under uncertain conditions remains a challenge [8]. Consequently, qualitative safety analysis ranges from individual functional module to entire autonomous vehicle system [9] were performed with the guidelines of related safety standards such as ISO21448 [10]. The identified challenges in autonomous driving safety examination can be complex requirements, non-deterministic algorithms, and fail-operational systems [11]. The established hazard identification techniques including Fault Tree Analysis (FTA), Event Tree Analysis (ETA), HAZOP, and STPA [12–14]. FTA and ETA focus on system failures and consequences, while HAZOP emphasizes process deviations and risks. STPA takes a broader system perspective, considering interactions and control failures. Nevertheless, research into linking the identified hazards to the ADS’s ODD and then formulate effective edge case scenarios remains ongoing.

### System Verification in Autonomous Driving

System verification ensures safety, reliability, and functionality, especially in mixed environments. Formal methods can offer a theoretically guaranteed playground for system verification and validation [15]. Fremont et al. [16] discussed transitioning from simulation-based testing to real-world applications, bridging the gap between theory and practice. Sun et al. [17] highlighted formal verification challenges in AI-driven systems. Despite their theoretical safety, formal methods face scalability issues, especially in dynamic environments and systems with learning components [18].

 Coverage-driven testing, can verify large systems effectively [19–22]. Brogle et al. [23] detailed verification of low-level hardware and software systems in a simulation environment. Wang et al. [24] emphasized the real-world scenario testing diversity to evaluate ADS robustness. Coverage-driven testing provides a structured approach for testing software systems, aiding in identifying gaps and measuring progress through metrics. However, there’s a risk of prioritizing quantity over quality, and achieving high coverage can be resource-intensive, potentially leading to a false sense of security.

### Test Case Generation

Beyond deriving scenarios from simulations or real-world events, researchers are exploring challenging adversarial scenarios [25]. Adversarial tests, easily produced in simulations, are often seen as a strategic game between the simulator and the system under test, aiming to challenge system performance [26]. Another method is combinatorial testing, which can detect failures within complex systems with a comprehensive array test suite generated by certain sampling mechanisms [27]. For more efficient testing, accelerated testing that subjects systems to edge cases to simulate long-term usage in a shorter period can be used [28]. However, in the context of ADS test case generation, these aforementioned methods face computational challenges, edge case identification challenges in sparse search spaces or limitations in testing real-driving systems due to the disparity between simulated and real scenarios, requiring additional alignment with operational profiles and safety constraints [29].

## Methodology

### Operation Flow Oriented HAZOP

**Fig. 1:**
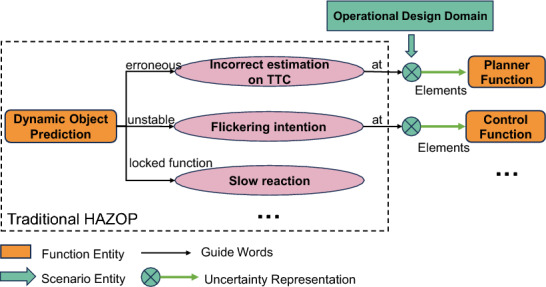
Example of operation flow-oriented HAZOP structure considering one function entity and its downstream functions

HAZOP is a systematic approach to identify hazards in complex systems widely used across industries for risk mitigation. It begins with system definition and selection of HAZOP guidewords like “more/less than intended”, “part of” or “erroneous” [30]. During HAZOP sessions, these guidewords are applied to the system’s components to uncover potential functional deviations. The traditional HAZOP approach, depicted as a black dashed box in Fig. 1, is relatively ineffective in modular risk assessment and lacks a clear method for addressing operability issues. A major ambiguity in this process is understanding how risks identified in one module might transfer to and impact downstream modules, including whether these risks can be effectively mitigated or not [12]. This could lead to an understatement of serious hazards not adequately addressed by downstream modules, or conversely, an overstatement of potential risks. The latter scenario can result in spurious findings and an unnecessarily expanded search space during the test case generation stage. Additionally, traditional HAZOP processes do not explicitly consider operability and its connection to the system’s ODD, which is a critical oversight in comprehensive risk assessment.

We propose incorporating operational flow and operating conditions into the hazard identification process, with added emphasis on the impact of learning-based functions. Our approach involves assessing failures that could introduce uncertainty to subsequent blocks relying on the output of a given functional entity. Building upon this, and guided by specific guidewords, we enhance hazard semantics with clauses such as “at operating condition *OC*, will affect elements, elem, in the scene have uncertainty representation $\sigma _{\text {{elem}}}$ post on the downstream function”. The scenario entities are elements in the given operating condition that will construct the test scenario attributes in the test case generation stage later in Sect. 3.3. We utilize the uncertainty representation $\sigma _{\text {{elem}}}$ for the prevalence of learning-based functions in modern autonomous systems. This uncertainty representation allows us to capture the stochastic behavior of these learning-based functions and conduct a more comprehensive hazard analysis of their cumulative effects.

**Fig. 2:**
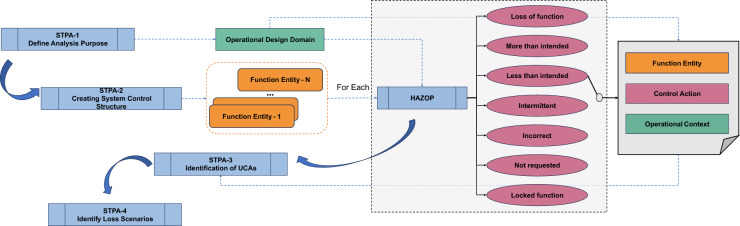
Illustration of cascaded STPA with HAZOP procedure

### Cascaded HAZOP and STPA

STPA is a model-based safety analysis technique tailored for complex systems. It transcends traditional safety analysis methods like HAZOP by considering not only component failures but also the intricate interactions and control dynamics within the system. Typically, it is structured into a four-step process:

**STPA-1 Defining Analysis Purpose**: This initial step involves outlining the ODD and identifying the abstract scenarios that the system must address.

**STPA-2 Creating System Control Structure**: Here, the system is modeled holistically, encapsulating functional relationships and interactions between entities, conceptualized as a network of feedback control loops.

**STPA-3 Identification of Unsafe Control Actions (UCA)**: This critical phase focuses on pinpointing UCAs within the system context that could potentially lead to hazardous scenarios.

**STPA-4 Identifying Loss Scenarios**: In the final step, for each UCA, two fundamental questions are addressed: “What failed?” and “What is the root cause of the failure?”. This approach provides a nuanced understanding of potential loss scenarios.

STPA sets itself apart by acknowledging that incidents in complex systems like ADS can occur even in the absence of component failures or human errors, stemming from a loss of control within the system. This perspective is particularly relevant for ADS, where the system’s complexity and interdependencies can lead to unforeseen hazards.

In this work, our objective is to merge operation flow-oriented HAZOP with STPA in a cascaded process. The strategy involves utilizing the modular feed-forward structure from each function entity in HAZOP to assist Steps 2 and 3 of STPA (as shown in Fig. 2). This information is then employed to configure the scenery and elements in test scenarios during the test case generation step (see Sect. 3.3). Once the system control structure is established in Step 2 of STPA, operation flow-oriented HAZOP is conducted for each function entity of interest.

HAZOP focuses on identifying and assessing potential deviations by examining what can go wrong at various points in a process or operation which is very detail-oriented. On the other hand, STPA takes a holistic view of system safety, analyzing the interactions between components and how they are controlled. After creating system structure in STPA-step2, we take each function process block in the system structure to the HAZOP process. We consider the following guide-words and their variation form on perception and planning, namely: loss of function, more than intended, less than intended, intermittent, incorrect, not requested, and locked function. For each process, the proposed HAZOP generates a list of potential hazards, operational problems, and the effectiveness of existing controls with the guided-words. For example, the perception work in “Less than intended” corresponding to larger object localization error (or increase in $\sigma _{\text {{elem}}}$) in the experiment; the “loss function” in planner functions would be stuck in the wrong state or previous plan path. The probability or severity of harm is evaluated based on the degraded element performance at its *OC*, for example, object detection performance drop in an open area without any object will have minimum affect on risk, whereas high risk in the crowded area. The list of potential hazards, operational problems generated from each HAZOP process will contribute to the STPA-3 to identify the UCAs with detailed context to facilitate the further test generation process.

**Fig. 3:**
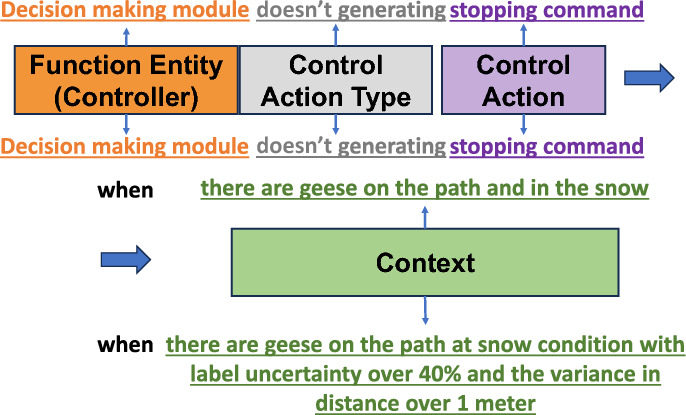
Example of UCA in the decision-making module facing perception degradation. The top section illustrates traditional STPA’s UCA, while the bottom section presents the proposed UCA obtained through the cascaded procedure, featuring a quantitative description to model the control action and its contexts

This approach allows the UCA identified in STPA Step 3 to be quantified, contrasting the traditional STPA methodology. Figure 3 demonstrates this comparison with an example that considers the performance of a low-level planner in relation to the perception system’s performance and environmental effects. The primary benefit of the proposed scheme lies in its ability to quantitatively represent performance degradation. This approach models the behavior of black-box, learning-based systems more effectively than using multiple discrete types of control actions and contexts. Recent STPA studies suggest that modeling a broader range of control actions can lead to the identification of more UCAs and, consequently, causal scenarios [14]. As demonstrated in Fig. 3, utilizing a quantitative representation to model performance degradation as the root cause of unsafe behavior proves more efficient in test case exploration compared to a limited, discrete set of scenario descriptions.

### Test Case Generation with Heterogeneous Search Space

In this section, we introduce a test case generation scheme that utilizes a heterogeneous search space derived from the cascaded HAZOP and STPA processes. This method entails identifying the root scenario and configuring both the discrete and continuous parameters for function entities and STPA elements, thereby constructing concrete test cases. Our approach prioritizes generating test scenarios as perceived internally by the ADS rather than creating an external driving environment.

The root scenario is an atomic scene within the ODD of the vehicle, encompassing basic stationary components such as road layouts, barricades, and signage [4]. A key advantage of utilizing the root scenario is its ability to limit the size of traffic and the number of dynamic objects involved. Covering every possible combination of pedestrians and vehicles on the campus is challenging, but it’s feasible to constrain this by focusing on specific atomic scenarios, such as a close region around an intersection.

**Fig. 4:**
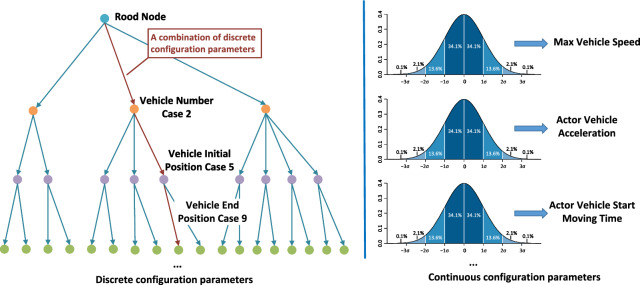
Example of generating test cases with discrete and continuous configuration parameters

Once the root scenario is established, we proceed to set up discrete configuration parameters layer by layer to generate concrete test scenarios. At the beginning of the test case generation process, ego-vehicle’s start and end positions are determined, while adversarial objects are randomly placed at different locations with varied paths to test the ADS’s responses. After setting up the manoeuvres and traffic participants, the next step involves exploring continuous search spaces such as related speeds, accelerations, and waiting times (as shown in Fig. 4). These continuous parameters can more accurately represent perception performance, especially in light of external factors that are typically challenging to model, like weather effects, communication delays, and localization errors. Afterwards, we superimpose human expert knowledge on driving scenarios to filter out unrealistic driving scenarios, for example removing u-turn, reverse driving maneuver which is forbidden in certain scenarios, or crowd crossing or stopping cases that has little exploration of system’s UCAs.

To summarize, based on STPA-1, we explore root scenarios based on the general ODD of the ADS. The discrete and continuous configurations are obtained in STPA-2, HAZOP, and STPA-3 to model the UCA context and potential hazards. Finally, loss scenarios are specially designed cases aimed at triggering faulty behavior in the system under test.

### Determine the Pass/Fail and Stop Criteria for Test Generation

Test case generation and safety validation primarily aim to verify if the system can handle scenarios as per performance criteria, and define operational boundaries for learning-based systems amidst stochastic behaviors and external factors [10]. Past criteria for test scenarios, derived from STPA-Step 4, focus on identifying and mitigating UCAs. A test case, depicted as a leaf node with continuous parameters in Fig. 4, fails if it erroneously deems a control action as safe.

To achieve the second goal, an exhaustive search on continuous configuration parameters, as shown in Fig. 4, becomes vital. The discrete configurations, derived from the atomic scenario, are finite and can be thoroughly covered. However, each leaf node will integrate continuous configurations to depict external factors. These parameters might include actors’ states (such as relative position and speed) and the ego vehicle’s perception of these states (mean and variance). For each nominal level of the continuous configuration set, coverage-driven testing can be employed to sample the nearby set of such levels. Applying the 3-sigma rule helps determine whether a configuration passes or fails. If it passes, the leaf node scenario with the chosen continuous configuration set is considered statistically 99.7% safe. Conversely, if it fails, it is documented as part of the “cannot handled scenario” for further testing and evaluation. As failure cases accumulate, a database is formed, encompassing both discrete and continuous parameters that define concrete operational scenarios beyond the system’s capabilities, effectively outlining the detailed ODD boundary. The test case generation process is deemed complete when the detailed ODD boundary reaches a point of convergence.

## Safety Validation of Autonomous Shuttle Vehicle: Case Study with WATonoBus

**Fig. 5:**
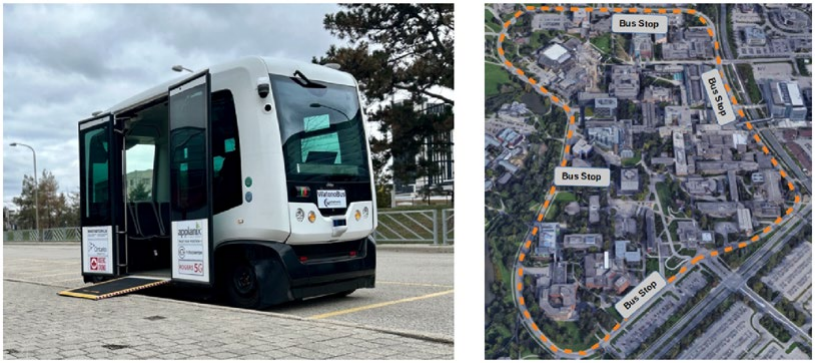
L2/L3 WATonoBus shuttle and its operation path on Ring Road [7]

To evaluate the efficacy of our proposed method in safety validation and test case generation, we conducted a case study using a real-world ADS. This study focuses on the WATonoBus, a low-speed autonomous shuttle service operating on Ring Road in the University of Waterloo campus, as depicted in Fig. 5. As detailed in Sect. 3, the initial application of our method involved performing a cascaded HAZOP and STPA analysis of the WATonoBus.

### STPA-1: Safety Analysis of WATonoBus System

The WATonoBus is an innovative L2/L3 autonomous shuttle, designed to address challenges in deploying autonomous vehicles in urban settings. The WATonoBus’s autonomous features must remain reliable and robust in various environmental and traffic conditions:

**Weather Conditions**: Sunny, rainy, snowy, and foggy.

**Traffic Participants**: Includes vehicles, motorcycles, pedestrians, and animals.

**Road Types**: Single driveways, all-way stop intersections, potential construction areas, bus stop zones, pedestrian crossings, and speed bumps.

Based on the system’s scope, safety analysis assumptions for the WATonoBus include: HD mapping for localization, assuming prior map knowledge; predominant driving scenarios: normal driving, intersection passing, and bus stop activities; operation at automation levels L2/3 with a human onboard for emergencies. Identified hazards include collisions, incorrect path following, and non-compliance with traffic rules.

### STPA-2: System Control Structure of WATonoBus

**Fig. 6:**
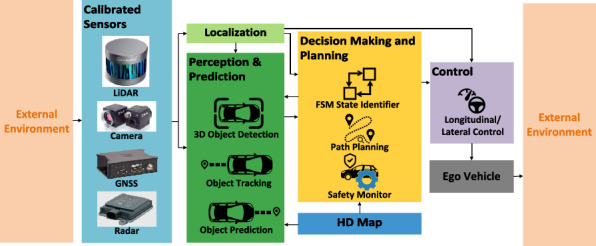
A schematic of the overall WATonoBus system

Figure 6 presents the high-level block diagram of WATonoBus system, featuring the following key functional modules:

**Localization module** handles ego vehicle positioning and captures static traffic features using dual techniques for redundancy: Applanix GPS sensor fusion algorithms ensure precise localization during normal driving, while a data-driven visual odometry method with LiDAR point cloud measurements provides basic localization during safety fallback strategies.

**Perception and prediction module** tracks surrounding traffic with integrated 2D camera, 3D LiDAR, and radar measurements. The side camera is activated during merging situations to execute the merging check algorithm.

**Decision making and planning module** manages tactical planning based on the surrounding environment. It employs a Finite State Machine (FSM) to determine the current driving state (normal driving, intersection passage, or bus stop pull-over/merging). For pull-over and merging, specific routes are planned to ensure safety. In normal driving, short-term ego path and velocity profile are iteratively planned based on drivable spaces and the ego vehicle safety performance is constantly monitored.

**Control module** employs both longitudinal and lateral Model Predictive Control (MPC). These controllers are finely tuned to track the planned path and velocity profile, providing desired inputs for the vehicle’s mechanical actuators.

**Table 1:** Hazardous Conditions of Prediction Function with HAZOP guidewords

#	Hazardous conditions	Description
PH1	Unable to adjust vehicle control autonomously	The ego vehicle cannot perceive or analyze its surroundings properly due to hardware or software malfunctions. It lacks the reference needed to adjust its motion in autonomous driving mode
PH2	Unable to proceed safety fallback strategy	The control mode arbiter cannot switch between autonomous and manual control, preventing the implementation of fallback strategies
PH3	Incorrectly adjust vehicle control autonomously	The ego vehicle struggles to adhere to traffic rules due to unreliable system hardware or software results. This impacts the vehicle’s ability to adjust vehicle dynamics in autonomous mode
PH4	Incorrectly adjust vehicle control autonomously	The ego vehicle struggles to execute safety strategies and maintain a safe state due to the suboptimal performance of the safety monitoring function in decision making module
PH5	Slow reaction to surrounding environment	One or more functional modules in the designed autonomous driving system can not efficiently process the algorithms and result in long latent time to the environment changes

### HAZOP Analysis of WATonoBus

As shown in Fig. 2, the HAZOP analysis begins by examining the system control structure to identify hazardous scenarios that may result from the failure of various functional modules. For each module, malfunctions and related hazardous conditions are determined using targeted HAZOP guidewords.

Following the methodology detailed in Sect. 3, a total of thirty-five malfunctions were identified across the five major functional modules previously mentioned. Among these, five primary vehicle-level hazardous conditions, which could potentially lead to vehicle crashes, are enumerated in Table 1.

### STPA-3: Identification of UCAs for WATonoBus

STPA pinpoints UCAs and associated design features. Referencing the system block diagram in Fig. 6, Table 2 lists the sub-controllers and their associated actions for WATonoBus.

**Table 2:** Control Actions of Sub-controllers in WATonoBus

Functional module (FM)	Context for control request	Control actions (CA)
FM1: Decision Making and Planning Module	General vehicle motion request at certain traffic scenarios	FM1CA1: Stop
		FM1CA2: Decelerate
		FM1CA3: Go
		FM1CA4: Pull over
		FM1CA5: Merge
FM2: Control Module	Lateral position adjustment request based on the planned trajectories.	FM2CA1: None
		FM2CA2: Lateral adjustment $\delta$ toward trajectory with steering angle modification
	Longitudinal position adjustment request based on the planned trajectories	FM2CA3: None
		FM2CA4: Longitudinal adjustment $\delta$ forward gas/brake pedal modification
FM3: Safety Strategy Selection in Decision Making Module	Safety state request based on vehicle status monitoring results	FM3CA1: Normal autonomous driving
		FM3CA2: Limited autonomous driving functionalities
		FM3CA3: Manual control
FM4: Control mode arbiter in control module	Vehicle operational mode confirmation request before implementing vehicle control	FM4CA1: Engage autonomous driving mode
		FM4CA2: Engage manual control mode

In conjunction with the primary vehicle-level hazardous conditions listed in Table 1, an additional set of guidewords is employed to identify unsafe control actions. These guidewords include: “not needed”, “Needed but intensity is incorrect”, “Needed but delivered incorrectly”, “Needed but start early or late”, and “No control actions provided but needed”.

Table 3 demonstrates the analysis conducted on the vehicle control module. For example, the highlighted Primary Hazard (PH), PH3, indicates a scenario where, despite only a lateral adjustment request and no longitudinal request, the module erroneously provides control commands for both directions, leading to an improper adjustment in vehicle control.

**Table 3:** Vehicle Control Functional Modules’ UCAs

Context for control request		Guide-words for assessing control actions				
Longitudinal position adjustment requests	Lateral position adjustment requests	Provided but not needed	Provided but more than intended	Provided but less than intended	Provided but too late	Not provided but needed
FM2CA3	FM2CA1	PH3	N/A	N/A	N/A	N/A
FM2CA3	FM2CA2	PH3	PH3	PH3	PH3, PH5	PH1
FM2CA4	FM2CA1	PH3	PH3	PH3	PH3, PH5	PH1
FM2CA4	FM2CA2	N/A	PH3	PH3	PH3, PH5	PH1

### STPA-4: Loss Scenarios and Causal Factors

Causal Factors (CF) for unsafe control actions are then identified by examining subsystems and their interactions. Understanding these relationships aids in generating test cases aimed at controlling, mitigating, or even eliminating unsafe control actions in the designed autonomous driving system.

**Fig. 7:**
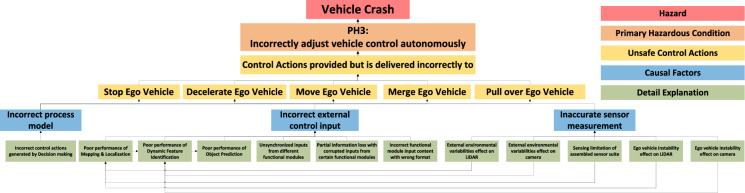
STPA Traceability Diagram Example of PH3

In this STPA study, the ultimate hazard, which all primary hazardous conditions will lead to, is a vehicle crash. This is followed by primary hazardous conditions, UCAs, and causal factors. A partial STPA traceability diagram with one branch under PH3 is shown in Fig. 7 as an example. This diagram serves as the basis for generating test cases and safety strategies to prevent corresponding hazardous conditions.

### Test Case Generation

**Fig. 8:**
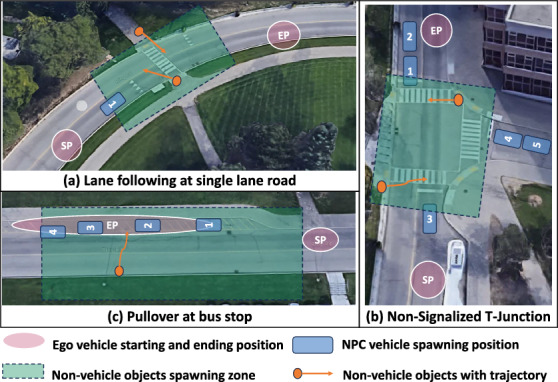
Example atomic scenarios for WATonoBus test case generation

The loss scenarios and the system’s ODD identified in the previous sections will be used for the test case generation process. In this section, we showcase the test case generation procedure for decision making and planning module focused on the three real-world atomic scenarios shown in Fig. 8. For real-world on-vehicle testing setup, readers can refer to Sect. 4.1 for detailed WATonoBus service environment and service scope. The demonstrated three atomic scenarios are identified through rosbag recordings during on-vehicle testing. However, more atomic scenarios can be created at a test field. The identified atomic scenarios are then reconstructed in MATLAB Driving Scenario Designer (DSD) using the proposed test case generation method. Following the Software-in-Loop (SIL) working principle, the generated test cases are used as input scenario files in a WATonoBus simulation platform, primarily built in Simulink. Specifically, a scenario reader block will interpret the test case scenario files and distributes the necessary scenario attributes to other WATonoBus software modules, including the decision-making and planning modules under test. After verifying these functional modules’ performance in simulation, they will then be validated in the same real-world scenarios to check the performance improvement.

#### Case 1: Lane Following at Crosswalk

In the root scenario of single-lane following, the driving task involves navigating from the Starting Point (SP) to the Ending Point (EP) in Fig. 8(a). As shown in Fig. 4, the first layer node specifies the number of other vehicle actors, limited to either zero or one. In the absence of other vehicles, the task simplifies to cruise mode. If present, even a single vehicle suffices for representing multiple vehicles, as the ADS of the ego vehicle primarily needs to focus on the vehicle directly ahead.

Assuming the discrete parameter tree from this root node includes three types of vehicles (car, truck, bus) and a maximum of two non-vehicle objects (choices of 0, 1, 2), the total potential leaf nodes amount to $$ _{1}{C}_{0}\times 3^0\times 3 +  _{1}{C}_{1}\times 3^1 \times 3 = 12$$, where $$ _{n}{C}_{r}$$ denotes the combination formula. Each leaf node explores continuous configuration parameters such as the vehicle’s maximum speed, moving time, and perception performance level. The actor vehicle’s speed is varied by 10 km/h increments up to 50 km/h. The moving time is uniformly sampled between 0 and 2 s, and the perception performance level relates to the noise level added to the ground truth object states. These parameters are used to generate concrete test scenarios, with detailed test metrics presented in Table 4.

The continuous search space in Table 4 demonstrate the richness of test scenario coverage in terms of the actors and the ego performance. To show the complete scenario generation process, the full coverage testing scenarios are listed in Table 4. The 3-sigma rule can be applied to the ego performance degradation configuration to further facilitate the testing process, based on the assumption that a failing process will not pass even more challenging scenarios. For example, when we gradually increase the perception noise in the continuous search space, one can identify the pass rate violating the requirement based on the 3-sigma rule at measurement error around 0.47 m from Table 4. In that case, further tests are not needed for the system under test as the stopping criteria is met. When comparing with the traditional coverage driven testing, at least 53% test cases are avoided in the perception noise search space.

**Table 4:** Summary of Test Cases Generated for the Three Atomic Scenarios and Their Validation Results

Case	Num of leaf nodes	Continuous search space	Num of erroneous leaves	Num of erroneous scenarios	Sample edge cases identified
1	12	- Start moving time: [0, 1.5]s	6	$133~(11.08\%)$	- Pedestrian zig-zag movement near traffic center line
					- Object detection degradation distance measure error $>0.47$ m
2	3072	- Perception noise: [0, 1]m	1130	$82443~(26.84\%)$	- Non-negotiate vehicle at pos4/5 when ego moving at the middle of the intersection
					- Pedestrian zig-zag movement within intersection area
		- Pedestrian sample velocity: [0, 1.2]m/s			- Detection degradation distance measure error $> 0.41$ m
3	512	- Vehicle sample velocity: [0, 5]m/s	276	$18895~(36.90\%)$	- Zig zag movement near curb
					- Stop region fully packed
					- Object vehicle with same speed continuously blocking the ego-vehicle

**Fig. 9:**
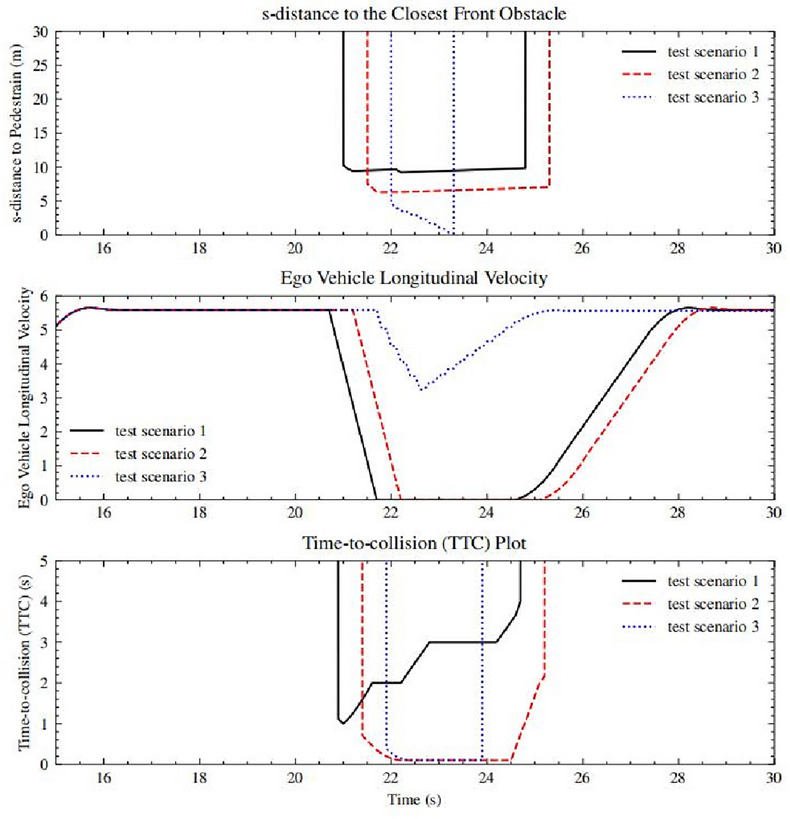
Example test results with three test scenarios for lane following task to validate WATonoBus decision-making module

Figure 9 showcases validation results for three lane-following test scenarios. The top row illustrates the s-distance in the Frenet frame between the pedestrian and the under-test ego vehicle, with a blue dashed line indicating a rule violation in test scenario 3. The middle and bottom rows display the ego vehicle’s velocity profile and overall TTC metrics, respectively. The black line (test scenario 1) represents a typical scenario where the ego vehicle meets expected performance standards. The red and blue dashed lines depict scenarios with intersected spatio-temporal trajectories (TTC close to 0) between the ego vehicle and the actor, each with distinct outcomes. In scenario 2, the ego vehicle stops before a collision due to the actor’s inability to react, lessening its severity. In contrast, scenario 3 is a failure case where the ego vehicle doesn’t slow down in time, resulting in a collision. Our method effectively addresses these situations by identifying long-tail cases.

Additionally, the bottom plot in Fig. 9 suggests that the WATonobus ADS’s effective braking TTC when encountering a sudden object is about one second. If the system detects an object within this time frame, it will probably lead to a failure, with the vehicle failing to decelerate in time and colliding with the actor, thus defining the ODD boundary.

#### Case 2: Intersection Handling at T-Junction

In the case of handling intersections, the ego vehicle must adhere to specific traffic rules: stop at all-way-stop signs; yield the right of way; avoid potential collisions; and stop for objects with a higher right-of-way intending to cross. These requirements, derived from STPA-1, serve as inputs for subsequent steps, as shown in Fig. 2.

For efficient test case generation during the testing setup stage, we consider the potential locations of other vehicles at five distinct spots, each possibly impacting the ego vehicle’s performance as UCAs. For instance, if there is only one other vehicle, its potential SP can vary from positions 1-5, as depicted in Fig. 8(b). With two potential vehicles, the combinations of vehicle positions at this level is $$ _{5}{C}_{2}$$. The general formula for calculating the total number of leaf nodes is as follows:

1 $$\begin{aligned} N_{\text {d}} = (N_{\text {ped}} + 1) \sum _{ {i}=0}^{N_{\text {s}}} ({N_{\text {s}}}{C}_{ {i}}\times N_{\text {type}}^{ {i}}) \end{aligned}$$

The number of low-speed pedestrians, the number of vehicle types, and the number of total vehicle position slots are denoted as $N_{\text {ped}}$, $N_{\text {type}}$, and $N_{\text {s}}$, respectively. Objects are not assigned to the opposite lanes near the ego vehicle’s SP, as they have no impact on the ego’s road rights and driving tasks. Vehicle positions 2 and 5 in Fig. 8(b) exemplify objects that might affect road rights at the All-Way-Stop due to the non-signalized intersection. This is particularly relevant considering the ambiguous right-of-way rule of “first-come-first-drive”, which could lead to potential conflicts in this root scenario.

In the T-Junction scenario, the starting positions of non-vehicle objects are sampled within the crossing’s covered area (green region in Fig. 8(b)). Their ending positions are first sampled, then filtered to determine whether they intersect with the ego vehicle’s trajectory. Test cases are generated to challenge the system under test; those with minimal impact on the ego vehicles are disregarded. As indicated in Table 4, the number of discrete parameter combinations increases exponentially with more slots and vehicle types. Fortunately, the number of test cases remains manageable due to the small size of the root scenario and a focus on major vehicle allocation patterns rather than random traffic level adjustments.

The testing procedure identifies 1130 out of 3072 discrete parameter configurations that lead to erroneous concrete scenarios. About 60% of the simpler cases are identified early, accelerating the testing process in the subsequent phase. Table 4 lists some identified edge cases, closely aligning with the traceability diagram in Fig. 7. Additionally, long-tail cases not identified by the STPA process also emerge. For example, a deadlock behavior at positions 4 and 5, where the ego vehicle gets stuck mid-intersection, is an undesirable outcome that the testing process helps to uncover.

#### Case 3: Pullover at Bus Stop

One crucial function of autonomous shuttling is to accurately pull over at designated locations to pick up passengers. While this maneuver resembles a lane change, it entails stringent requirements such as avoiding curb and side obstacle collisions and dynamically selecting the final location based on the surrounding dynamic environment.

As depicted in Fig. 8(c), the SPs of actor vehicles are randomly allocated within the ego vehicle’s EP zone. Task compliance is evaluated based on successfully pulling over to the designated stopping region and leaving adequate space for subsequent merging maneuvers.

Utilizing Eq. (1), we identified a total of 512 discrete branches. Test cases are generated by delving into continuous parameter configurations, with one hundred concrete test cases uniformly sampled for each discrete node. The detailed validation results are presented in Table 4. Notably, the proportion of erroneous scenarios exceeds that of the T-Junction case, despite fewer actors in the configuration space. Our test generation and validation process revealed that certain scenario contexts might lead to violations of predefined rules. For instance, as the number of vehicles increases, the stop region may become overcrowded. An illustrative scenario involves an actor vehicle moving alongside the ego vehicle, continuously blocking the stopping area, thus rendering the predefined task unsuccessful in these specific scenarios. These scenarios extracted through our proposed approach are invaluable for the ongoing development of the WATonobus ADS software stack.

## Discussion

Developing an understanding of the true capabilities and limitations of modern ADS is crucial, especially for real-world systems. This knowledge is garnered by testing these systems in various scenarios to assess their capabilities, leading to an informed safety state or refined ODD. Complex modern ADS, which often incorporate state-of-the-art learning-based technologies, necessitate novel safety verification and test case generation strategies. Hazard-based testing remains a proven and effective tool for identifying scenarios where these systems would fail [12].

This paper introduces a cascaded STPA and HAZOP process for identifying potential hazards and their causal relationships, creating test cases, and refining operational boundaries. Our method generates test cases by expanding a tree from the root node, parameterizing discrete configurations, and sampling from various continuous parameter sets. The STPA-HAZOP process guides this expansion, with the UCA enhanced by specific, formal contexts and highlighted causal factors from Steps 3 and 4. This extended context is crucial for generating and grouping long-tail test cases that are not adequately addressed by the system under test.

We applied this methodology to a low-speed autonomous bus used for campus shuttling. While STPA identifies known risks, its key advantage lies in uncovering new, unforeseen scenarios-the “unknown unknowns”. Thus, our hazard-based scenarios, augmented by STPA, enhance requirement-based testing methods, increasing the scope of “known”. Test cases, originating from atomic scenarios, incrementally increase in complexity, aligning the test domain with the system’s real ODD. Our method, unique in using the root scenario for automatic grouping and critical scenario identification, complements a robust, efficient hazard-based framework for ADS safety evaluation.

In our test case generation, the configured continuous search space aims for a responsibility-free state, offering a narrower search scope than a collision-free state. For instance, in the bus-stop pullover scenario, we assume vehicles don’t reverse, a rule not always followed in reality. However, passing all scenarios in this configuration likely ensures responsibility-free driving function performance. Sensor uncertainty failures also aid in developing the ADS’s decision-making and safety monitoring modules. Future extensions of this study will consider critical modern transportation factors: traffic rules, driving styles, and societal behaviors. Encoding traffic rules enhances test case realism, while quantifying and adjusting driving styles and societal behaviors within the test generation search space will cover more edge cases, particularly those arising from other traffic participants’ misbehavior. Furthermore, accurately modeling continuous parameters such as object detection noise based on real-world data would prove beneficial. This approach could result in a more precise representation of cumulative effects on other modules and the generation of more realistic edge case scenarios.

## Conclusion

The research successfully demonstrates a novel approach for safety verification in autonomous vehicles, particularly focusing on the real-world L3 autonomous shuttling service vehicle, WATonoBus. By combining HAZOP and STPA, it provides an effective framework for addressing the complex safety challenges in autonomous transportation. The proposed safety analysis procedure and the test generation approach are efficient for verifying system safety boundaries and identifying edge cases for continuous engineering. The real-world application of WATonoBus highlights the practicality and effectiveness of these methods, contributing significantly to the field of autonomous vehicle safety and offering valuable insights for future research and development in this rapidly evolving domain. In the future, we would like to further extend this study to various ADS systems including truck and connected vehicles and check the potential applications in fast development for these systems’ safety operational domain specification.
